# Vascular Function in Norwegian Female Elite Runners: A Cross-Sectional, Controlled Study

**DOI:** 10.3390/sports10030037

**Published:** 2022-03-02

**Authors:** Karoline Holsen Kyte, Trine Stensrud, Tore Julsrud Berg, Ingebjørg Seljeflot, Jonny Hisdal

**Affiliations:** 1 Institute of Clinical Medicine, Faculty of Medicine, University of Oslo, 0318 Oslo, Norway; t.j.berg@medisin.uio.no (T.J.B.); uxinlj@ous-hf.no (I.S.); jonny.hisdal@medisin.uio.no (J.H.); 2 Department of Vascular Surgery, Oslo University Hospital, Aker, 0586 Oslo, Norway; 3 Department of Sports Medicine, Norwegian School of Sport Sciences, 0806 Oslo, Norway; trines@nih.no; 4 Department of Endocrinology, Morbid Obesity and Preventive Medicine, Oslo University Hospital, Aker, 0586 Oslo, Norway; 5 Center for Clinical Heart Research, Department of Cardiology, Oslo University Hospital, Ullevål, 0424 Oslo, Norway

**Keywords:** relative energy-deficiency in sport, female athlete triad, female endurance athletes, flow-mediated dilatation, carotid intima-media thickness, carotid-femoral pulse wave velocity

## Abstract

In general, aerobic exercise has a positive impact on the vascular system, but the syndrome of relative energy-deficiency in sports (RED-S) makes this impact less clear for the athlete. The present cross-sectional controlled study aimed to investigate the vascular function in female elite long-distance runners, compared to inactive women. Sixteen female elite long-distance runners and seventeen healthy controls were recruited. Assessments of vascular function and morphology included endothelial function, evaluated by flow-mediated dilatation (FMD), vascular stiffness, evaluated with pulse wave velocity (PWV), carotid artery reactivity (CAR %), and carotid intima-media thickness (cIMT). Blood samples included hormone analyses, metabolic parameters, lipids, and biomarkers reflecting endothelial activation. RED-S risk was assessed through the low energy availability in female questionnaire (LEAF-Q), and body composition was measured by dual-energy X-ray absorptiometry (DXA). We found no significant differences in brachial FMD, PWV, CAR %, cIMT, or biomarkers reflecting endothelial activation between the two groups. Forty-four percent of the runners had a LEAF-Q score consistent with being at risk of RED-S. Runners showed significantly higher HDL-cholesterol and insulin sensitivity compared to controls. In conclusion, Norwegian female elite runners had an as good vascular function and morphology as inactive women of the same age.

## 1. Introduction

Regular endurance training and increased aerobic capacity are generally accepted to have a positive influence on the cardiovascular system, by modifying classical risk factors associated with cardiovascular disease [1]. These changes include a more favorable lipid profile, increased insulin sensitivity, and a positive impact on blood pressure. However, previous studies have indicated that in healthy young women, it is not entirely clear how large amounts of endurance training affect cardiovascular health [2,3,4].

The number of women participating in endurance sports competitions has increased in the last decades [5]. Gender-specific injuries and medical conditions unique to the female athlete exist and should be given attention [6]. Among professional female endurance athletes, the “female athlete triad”, currently renewed and replaced by the broader term “relative energy deficiency in sports” (RED-S), is considered an important challenge for the athletes’ health [7]. The fact that male athletes are also affected, and the complexity involved in the syndrome, are the reasons for this broader term. The syndrome of RED-S refers to an “impaired physiological functioning caused by relative energy deficiency”, and includes, but is not limited to impairments of metabolic rate, menstrual function, bone health, immunity, protein synthesis, and cardiovascular health [8]. Although men are included in this term, there are still conditions that apply specifically to women [6]. 

Impaired cardiovascular health includes unfavorable lipid profile and endothelial dysfunction, both previously shown in amenorrheic athletes [9,10]. Amenorrhea is associated with low levels of estradiol, a hormone playing an important role in preventing cardiovascular diseases by stimulating the production of nitric oxide (NO), which causes vasodilation [11]. 

Thus, RED-S has several negative health consequences, including effects on the vascular system. Traditionally, vascular function is measured with flow-mediated dilatation (FMD) and carotid response, while morphological changes are evaluated with pulse wave velocity (PWV) and intima-media thickness (IMT). Despite previous studies demonstrating a reduced FMD-response in female athletes with RED-S [9,10], little is known how long-distance running or RED-S affects carotid response, IMT, and PWV. The nature of running, i.e., striving towards a lean body figure, makes long-distance runners particularly vulnerable to RED-S. Hence, there is a need for an increased understanding of vascular health in female elite runners, beyond the endothelial function. 

The primary objective of the present study was therefore to investigate the vascular function and morphology in Norwegian female elite long-distance runners, including the endothelial function, compared to inactive women. Based on previous studies, we hypothesized reduced vascular function in elite runners compared to inactive women.

## 2. Materials and Methods

Written information about the study was distributed to Norwegian female runners in the top 20 of the long-distance running statistics lists in Norway 2019, as well as announced on social media. Recruitment focused on long-distance running, including trail running. The inclusion criteria were as follows: age 18–35 years, healthy, non-smoking, nullipara, and a minimum of 8 h endurance training weekly. A control group of inactive women was recruited among students at the University of Oslo. They were screened using the same inclusion criteria as for the runners, except that the amount of training was restricted to a maximum of 2 h weekly. The study was approved by the Regional Committees for Medical and Health Research Ethics (REC South East, reference 2019/155). After written informed consent, 16 endurance-trained women and 17 inactive women were recruited between October 2019 and January 2020. No regular medication, except oral contraceptives, were accepted. Six of the athletes and five of the inactive controls were taking oral contraceptives.

Prior to testing, all participants received information about the study, including questions regarding their menstrual cycle and the use of oral contraceptives. This allowed us to time the test day for vascular examinations and blood tests to the menstrual cycle. 

The study was conducted in three phases. In phase one, assessments of vascular function and morphology were performed, including endothelial function, evaluated by flow-mediated dilatation (FMD), vascular stiffness, evaluated with pulse wave velocity (PWV), carotid artery reactivity (CAR %), and carotid intima-media thickness (cIMT). Participants also answered the low energy availability in female questionnaire (LEAF-Q) at the same session. In the second phase, all blood samples were collected. In phase three, the participants performed dual-energy X-ray absorptiometry (DXA) and a VO_2_max test.

Prior to the vascular examinations, the participants were asked to arrive in a fasting state (>6 h) and abstain from caffeine and alcohol consumption for the last 24 h, and to avoid intense physical activity or consuming vitamin C for the last 12 h. Assessments were performed between day 1 and 7 of the menstrual cycle to avoid the influence of circulating estradiol [12]. 

After 15 min supine resting in a climate-controlled, quiet room, resting blood pressure was measured, followed by analyses of vascular function and vascular morphology. During the vascular examinations, heart rate was continuously monitored with a 3-lead ECG to enable ECG-gated acquisition of the ultrasound images. 

Endothelial function was assessed by FMD in arteria brachialis, in the right arm, according to current consensus guidelines [12]. Baseline diameter was recorded for 60 s before cuff inflation. To induce reactive hyperemia, a standard blood pressure cuff (Boso arm-cuff, cuff size 22–32 cm) was placed on the lower right arm distally to the ultrasound probe, two inches below the ante-cubital fossa. The brachial artery was imaged 5–9 cm above the ante-cubital fossa. The cuff was inflated for 5 min with pressure at 230 mmHg, and the post deflation diameter was monitored continuously for the next 3 min. 

Brachial artery diameter and blood flow were measured using a 9 MHz linear transducer connected to an ultrasound machine (Vivid E-95, GE Vingmed Ultrasound, Horten, Norway). An insonation angle < 60° was used to assess correct velocity measurements. A custom-made mechanical arm ensured that the transducer remained in a fixed position over the right brachial artery throughout the test.

Data analyses of the ultrasound images were performed using a custom-designed edge-detection and wall-tracking software (Brachial Analyzer, Medical Imaging Applications LLC, Coralville, IA, USA), with automatic measures of arterial diameter. The mean diameter from 10 s before cuff deflation was used as baseline-diameter, and FMD was defined as the maximum percentage change in artery diameter from baseline to maximal vasodilation during the test.

Participants were in a supine position for assessment of the carotid artery reactivity to sympathetic activation (CAR %). The left carotid artery was recorded continuously for 30 s before the participant was instructed to put their left hand in a bucket of ice slush for 90 s, with continuous assessment of the carotid artery using a 9 MHz linear transducer connected to an ultrasound machine (E-95, GE Vingmed Ultrasound, Horten, Norway). To ensure high quality and to avoid interference in the images, participants were asked to breathe normally and not speak during the measurement [13]. The mean of the diameter measured every 10th s for the first 30 s was defined as the baseline diameter. To capture the maximal change in diameter, the carotid artery diameter was measured every 10th s during the 90 s cold pressure test. Finally, CAR % was calculated as the relative change from baseline diameter to peak dilation or construction during the test [13].

Intima-media thickness (cIMT) was measured with ultrasound in a supine position (E-95, GE Vingmed Ultrasound, Horten, Norway). Measurement of cIMT was performed on a 1-cm long segment of the far wall of the left common carotid artery, about 1 cm proximal to the carotid bulb, using semi-automated edge detection, analyzed by integrated software on the ultrasound machine.

Carotid-femoral pulse wave velocity (cfPWV) was recorded transcutaneously at the right carotid and right femoral artery, using applanation tonometry (SphygmoCor apparatus, Atcor, West Ryde, Australia). Two different pulse waves were obtained at each site. The recordings were selected according to predetermined requirements in the software [14], and the average of two recordings was used in the analyses.

LEAF-Q was answered by all study participants and used as a screening tool to identify female athletes at risk for long-term low energy availability and RED-S [15]. A total score of ≥8 is normally considered at risk of the RED-S.

Blood samples were collected from a peripheral vein in a resting state between 08:00 a.m. and 10:00 a.m. after an overnight fast and prior to exercise. Sample collection performed once during the menstrual cycle day 1–7, included hormone analyses (estradiol, luteinizing hormone (LH), follicle-stimulating hormone (FSH), testosterone, sex hormone-binding globulin (SHBG), thyroid-stimulating hormone (TSH) and free thyroxine (fT4)), metabolic parameters (insulin, C-peptide, glucose, and glycated hemoglobin A1c (HbA1c)), and lipids (total cholesterol, high-density lipoprotein (HDL)-cholesterol, low-density lipoprotein (LDL)-cholesterol, triglycerides, apolipoprotein A, and apolipoprotein B).

Total cholesterol, HDL, LDL, triglycerides, apolipoprotein A, apolipoprotein B, and HbA1c were measured by routine conventional methods. Hormone analyses were performed at the Hormone Laboratory, Oslo University Hospital. The laboratory is accredited by the Norwegian Accreditation according to the requirements of the NS-EN ISO/IEC 17025, TEST 099. Serum samples were analyzed for TSH, fT4, estradiol, LH, FSH, testosterone, SHBG, insulin, and C-peptide. TSH was measured with non-competitive immunofluorometric analysis by Autodelfia (Wallac Oy, Turku, Fin-land), and fT4 was measured with solid-phase time-delayed fluoro-immunoassay with back-titration by Autodelfia (Wallac Oy, Turku, Finland). Estradiol was determined using chemiluminescence immunoassay (CLIA). The concentrations of FSH, LH, and SHBG were determined using a non-competitive immunoluminometric assay (ILMA). C-peptide and insulin were analyzed using non-competitive electrochemical luminescence immunoassay (ECLIA) and testosterone was determined using liquid chromatography-tandem mass spectrometry (LC-MS/MS). Analytical coefficients of variation (CVa): TSH, 3%, fT4, 5%, estradiol, 11%, FSH, 7%, LH, 9%, SHBG, 7%, insulin, 7%, C-peptide, 4%, testosterone, 12%.

Insulin resistance (IR) was calculated by the method of homeostasis model assessment 2 (HOMA2), using the HOMA2-calculator.

In addition, blood samples for biomarkers reflecting inflammatory, hemostatic, and vasoactive endothelial activation were collected at the same visit. Serum was prepared within one hour by centrifugation at room temperature at 2500× *g* for 15 min and EDTA and citrated blood was stored on ice until centrifugation within 30 min at 2800× *g* for 20 min. All samples were frozen at −80 °C until analyses. Commercial ELISA kits were used for vascular cell adhesion molecule 1 (VCAM-1), intracellular adhesion molecule 1 (ICAM-1), E-selectin, P-selectin (R&D Systems Europe, Abingdon, Oxon, UK), von Willebrand Factor (vWF) (Asserachrom vWF Ag, Stago Diagnostica, Asnieres, France) and high sensitivity CRP (DRG Instruments, Marburg/Lahn, Germany). Intra assay coefficients of variations (CVs) were 3.3%, 2.1%, 6.5%, 3.9%, 9.5%, and 4.3%, respectively. L-arginine, asymmetric dimethylarginine (ADMA) and symmetric dimethylarginine (SDMA) were determined by high-performance liquid chromatography (HPLC) and precolumn derivatization with o-phthaldialdehyde (OPA) (Sigma Chemicals Co, St Louis, MO, USA). CVs were 5.9%, 7.0%, and 9.6%, respectively.

The maximal oxygen consumption (VO_2_max) was conducted by using a breath-by-breath gas analysis system (OxyconPro analyzer; Jaeger, Würtzburg, Germany) on a treadmill (ELG 90/200 Sports; Woodway, Weil am Rhein, Germany). After a 15 min warm-up period, the incline was increased to 5%. Output speed was adjusted to the participant’s fitness level, and the speed was thereafter increased by 1 km/h every min until exhaustion. VO_2_max was defined as the mean of the two highest 30 s measurements and in addition, respiratory exchange ratio (RER) > 1.1.

Height and body weight were measured prior to the DXA scan. DXA was performed (Lunar, Prodigy Densitometry, GE Medical Systems, Chicago, IL, USA) to determine the body composition.

Statistical analyses were performed in SigmaPlot 14.0 (Systat software Inc., San Jose, CA, USA). The normality test (Shapiro-Wilk) failed for some of the variables, and data are therefore presented as median (25th–75th percentile) throughout. The data present in this study are openly available in the Supplementary Materials (Supplementary Table S1). Non-parametric statistics (Mann–Whitney Rank Sum Test) were used to test for significant differences between the groups. A value of *p* < 0.05 was accepted statistically significant.

## 3. Results

### 3.1. Participants

Participants’ characteristics are presented in Table 1. The median weekly running distance in the runners’ group was 120 km (110–120). A total LEAF-Q score ≥ 8 was found in seven of the runners (44%). Three of the runners (19%) reported that they had secondary amenorrhea, defined as no menstrual bleeding for the last 3 months. There were no runners with a history of primary amenorrhea.

### 3.2. Vascular Function and Vascular Morphology

**Figure 1:**
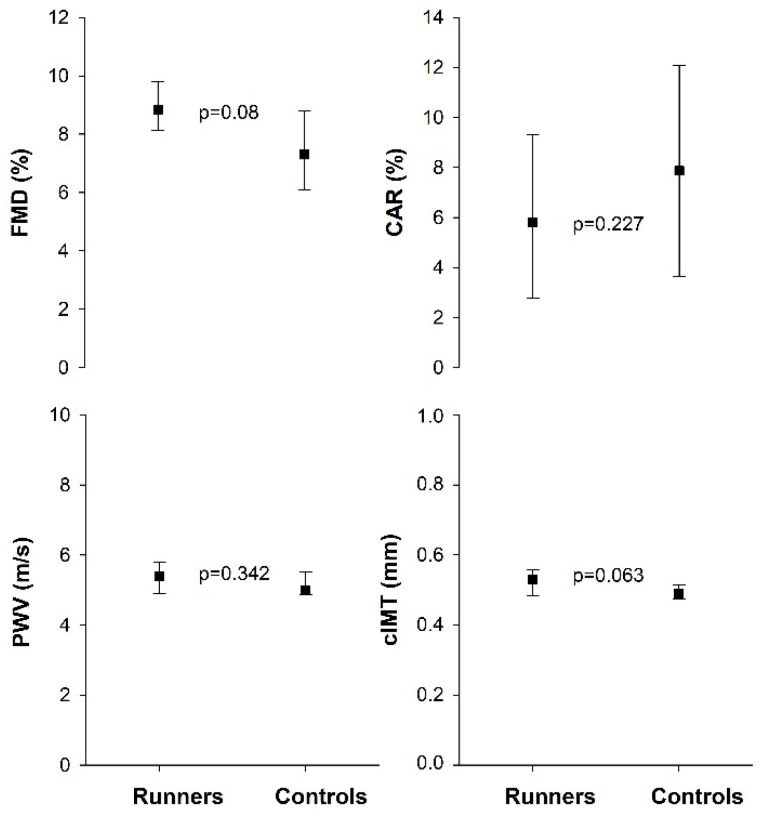
Flow-mediated vasodilatation (FMD), carotid artery reactivity (CAR), pulse wave velocity (PWV), and carotid intima-media thickness (cIMT) in runners (n = 16) and controls (n = 17). Values are presented as median (25th–75th percentiles).

Baseline diameter of *a.brachialis* was similar between runners and controls, *p* = 0.665 (3.48 mm (3.10–3.66) vs. 3.35 mm (3.03–3.64), respectively). Further, there was no significant difference in FMD, cIMT, CAR %, or PWV between the two groups (Figure 1). Runners had higher HDL-cholesterol and Apolipoprotein A levels than the inactive controls (*p* = 0.017 and 0.027, respectively). Nevertheless, both groups showed lipid values within the reference range (Table 2). There was no significant difference between the groups in any of the biomarkers reflecting endothelial activation (Table 2).

### 3.3. Endocrine and Metabolic Variables

Endocrine and metabolic characteristics for the study groups are shown in Table 3. Despite lower levels of LH for the runners (*p* = 0.010), there were no significant differences in hormone levels between groups. Insulin, C-peptide, and HOMA-IR were lower in runners compared to the inactive controls (*p* = 0.022, 0.052 and 0.025, respectively).

## 4. Discussion

There is limited knowledge of how regular endurance training affects vascular function and vascular morphology in female athletes. To our knowledge, this is the first study assessing vascular function and vascular morphology among female elite runners. The main finding of the present study was that Norwegian female elite long-distance runners had well-preserved vascular function, compared to age-matched inactive women. No significant differences in either functional or morphological measurements, as well as on biomarkers of endothelial activation and function were observed between the study groups.

The runners in our study are very well trained and had an average VO_2_max at 64.3 (mL·kg^−1^·min^−1^), nearly 50% higher than the inactive group. Aerobic exercise is well known to improve endothelial function when reduced by age, cardiovascular diseases, hypertension, or type-2 diabetes [16,17], while an equally positive effect cannot be expected when the endothelial function is well preserved at baseline as in our study groups.

Brachial artery FMD is considered the gold standard for assessing vascular function and was therefore used as the site for FMD measurements despite participants being endurance-trained runners. The popliteal artery may also have been considered, but to be able to compare with other studies, we have chosen the most common method. In the present study, we did not find any significant difference in FMD in the brachial artery between the two groups, which corresponds to the study of Moe et al. [3], who showed similar FMD in trained and sedentary females. Similarly, Rickenlund et al. [9] showed equal FMD-response in eumenorrheic athletes compared to sedentary, while FMD was impaired in amenorrheic athletes. More studies have been conducted on male athletes, reporting both similar [18,19], and improved [2,20], FMD-response compared to inactive controls. However, our observations support that high-level running performance seems not to be unbeneficial for the athletes in terms of endothelial function. Pyke et al. [21] demonstrated an inverse relationship between peak shear rate and baseline diameter of the brachial artery during a reactive hyperemia test. In that study, baseline diameter varied from 3.3 to 5.3 mm. In the present study, we did not observe any difference in baseline diameter of *a.brachialis* between the runners and controls, and also small variation in brachial baseline diameter between the runners and controls (3.48 mm (3.10–3.66) vs. 3.35 mm (3.03–3.64), respectively). It is therefore not likely that differences in diameter or shear rate may have influenced the FMD results in the present study.

Further, no differences between groups were observed in CAR % or PWV. CAR % represents a novel test of vascular health in relation to cardiovascular disease and a supplement to structural measures of the carotid artery. CAR % reflects coronary artery vasodilator function, and impaired CAR % has been observed among older individuals and persons with cardiovascular risk factors [22]. To our knowledge, this study is the first to investigate CAR % in young female endurance athletes.

Previous studies on arterial stiffness (PWV) in endurance athletes have shown divergent results [23,24]. Our observations correspond to the findings of Bjarnegård et al. [25] who showed no difference in carotid-femoral PWV between runners, sedentary controls, and normally active controls. Beyond this, few studies have compared vascular stiffness in runners and inactive controls. However, some interesting associations are shown; Namgoong et al. [24] did not observe any relationship between arterial stiffness and aerobic capacity in healthy young women and men, while an inverse relationship between cardiorespiratory fitness and augmentation index, a surrogate marker of arterial stiffness, was observed by Denham et al. [26]. In contrast, marathon running has been linked with higher arterial stiffness [23]. Further, an inverted-U shape dose-response curve has been suggested for the exercise-related benefits in arterial stiffness [23], which may explain the divergent results. As far as we know, there are no previous studies on vascular stiffness as a possible marker for RED-S. However, the cardiovascular complications of RED-S are similar to what we see in patients with anorexia nervosa. Previous research has shown significantly increased vascular stiffness (PWV) in patients with anorexia nervosa compared to healthy controls [27], indicating that patients with anorexia nervosa may be at increased risk for further cardiovascular diseases. However, it remains unexplained whether these results may be transferable to amenorrheic athletes/athletes suffering from the syndrome of RED-S.

Increased cIMT is considered an early sign of atherosclerosis. Our observation of no significant difference in cIMT between runners (0.53 mm) and controls (0.49 mm) is in accordance with previous cross-sectional studies on female athletes [19,25]. Bjarnegård et al. [25] showed similar cIMT in female runners, sedentary controls, and normally active controls. Similarly, Bittencourt et al. investigated cIMT in professional half-marathon runners, and found no difference between female athletes and controls, while male controls had higher cIMT compared with male athletes [19]. To our knowledge, there are no previous studies on intima-media thickness as a possible marker for RED-S. Interestingly, previous research has indicated no differences in cIMT between patients with amenorrhea due to anorexia nervosa, compared with healthy controls [28].

Further, we did not observe any differences between the study groups in biomarkers of endothelial activation, neither related to inflammatory, thrombotic, or vasoactive components, corresponding to the lack of difference in functional vascular measurement, and according to the study of Rickenlund et al., who showed no difference in VCAM between athletes and sedentary controls (9). However, previous research on endothelial activation in endurance athletes has mainly focused on the acute effect of exercise on endothelial activation, limiting the possibility of comparison.

In the present study, 44% of the runners had a LEAF-Q score ≥ 8, consistent with being at risk for RED-S [29]. Long-distance running is energy-intensive, and performance is also weight-dependent, which explains the increased risk for RED-S in the study group. However, it is important to emphasize that LEAF-Q is only a screening tool to identify individuals at high risk of low energy availability and RED-S, vs. low risk, respectively, and therefore cannot be used to determine whether the runners actually have low energy availability or not.

Suppressed sex hormones and functional hypothalamic amenorrhea (FHA) are part of the syndrome of RED-S [7,29]. FHA has previously been reported among 60% of elite female long-distance runners [30,31,32]; consequently, we had expected a high prevalence of FHA in our study. With only three athletes (19%) reporting amenorrhea, our population seems to be quite healthy compared to prevalence estimates from earlier research [30,31,32]. However, runners had lower LH compared to controls and had lower estradiol though not significant, thus we cannot rule out subclinical hypothalamic disturbances, a potential indicator for energy deficiency [32].

The claim of a quite healthy runner population in our study is supported by other findings, i.e., higher levels of HDL, and similar beneficial results for the other lipids. This is quite the opposite of what is associated with a hypogonadal state, such as in amenorrheic athletes [9,33]. A further positive sign is increased insulin sensitivity in runners compared to controls. This might be explained by training, increased VO_2_max, and lower fat mass in runners.

The relatively small sample size is a limitation of this study. However, there are a limited number of female elite runners in Norway. Another possible limitation may be the use of oral contraceptives, which may hide an underlying menstrual disturbance [34]. However, a large proportion of the best runners use hormonal contraception [34]. Our population is thus representative, and by excluding them, it would be difficult to find a sufficient number of runners to participate in the study. Finally, we have no information on the family history of CVD, but it is unlikely that this will have an impact on our findings.

## 5. Conclusions

Norwegian female elite runners had an as good vascular function and morphology as inactive women of the same age, including both endothelial function, vascular stiffness, vascular morphology, carotid artery reactivity, as well as biomarkers reflecting endothelial activation. The low prevalence of amenorrhea among the runners in our study may have contributed to this. More research, with larger sample sizes, is needed to fully understand how elite-level running affects vascular function and vascular morphology in women, beyond endothelial function, which has already been truly investigated.

## Figures and Tables

**Table 1:** Clinical characteristics in runners and control group.

Groups	Runners (n = 16)	Controls (n = 17)	*p*-Value
Age (yr)	27.0 (24.3–30.0)	26.0 (24.0–27.5)	0.196
Height (cm)	169 (164–176)	172 (166–178)	0.328
Weight (kg)	55.9 (54.3–60.7)	63.0 (60.8–70.2)	<0.001
BMI (km/m^2^)	19.9 (18.9–21.0)	22.0 (20.6–24.0)	<0.001
SP (mmHg)	110 (110–118)	110 (110–115)	0.789
DP (mmHg)	70 (70–78)	70 (70–70)	0.542
VO_2_max (mL·kg^−1^·min^−1^)	64.3 (62.5–66.7)	44.8 (41.8–45.4)	<0.001
Endurance training (hrs·wk^−1^)	11.0 (9.0–14.5)	1.0 (0.0–1.0)	<0.001
Fat mass (%)	16.9 (15.3–19.0)	29.7 (25.6–33.7)	<0.001
Fat mass (kg)	9.1 (8.2–10.7)	19.3 (16.1–21.9)	<0.001
LEAF-Q (total score)	7.0 (4.3–9.0)	3.0 (1.0–6.0)	0.004

Values are expressed as median (25th–75th percentiles). Abbreviations: BMI, body mass index; SP, systolic blood pressure; DP, diastolic blood pressure; LEAF-Q, low energy availability in female questionnaire. *p*-values refer to difference between groups.

**Table 2:** Serum lipids and biomarkers reflecting endothelial activation in runners and control group.

Groups	Runners (n = 16)	Controls (n = 17)	*p*-Value
Total cholesterol (mmol/L)	4.4 (4.0–5.125	4.0 (3.6–4.6)	0.101
HDL cholesterol (mmol/L)	1.9 (1.7–2.4)	1.5 (1.4–1.9)	0.017
LDL-cholesterol (mmol/L) Ratio total cholesterol/HDL	2.4 (1.9–2.9) 2.2 (2.1–2.5)	2.3 (1.9–3.0) 2.5 (2.2–2.8)	0.900 0.101
Triglycerides (mmol/L)	0.7 (0.5–1.0)	0.8 (0.6–1.050	0.446
Apolipoprotein A	1.82 (1.58–2.04)	1.49 (1.37–1.75)	0.027
Apolipoprotein B	0.73 (0.65–0.94)	0.73 (0.61–0.88)	0.914
VCAM (ng/mL)	695 (607–784)	707 (634–817)	0.577
ICAM (ng/mL)	200 (188–215)	195 (178–215)	0.552
CRP (mg/L)	0.31 (0.18–0.53)	0.57 (0.22–1.79)	0.097
E-selectin (ng/mL)	25.4 (18.8–39.2)	30.9 (19.9–38.4)	0.914
vWF (%)	93 (77–108)	82 (70–90)	0.084
P-selectin (ng/mL)	21.9 (20.7–25.8)	21.5 (19.1–24.7)	0.321
L-arginin (µM)	36.0 (30.5–41.1)	30.6 (28.9–40.0)	0.322
ADMA (µM)	0.36 (0.32–0.38)	0.35 (0.32–0.38)	0.677
SDMA (µM)	0.23 (0.18–0.24)	0.20 (0.19–0.21)	0.163
Ratio L-arg/ADMA	101 (89–114)	94 (88–111)	0.482

Values are expressed as median (25th–75th percentiles). *p*-values refer to difference between groups.

**Table 3:** Endocrine and metabolic variables in runners and control group.

Groups	Runners (n = 16)	Controls (n = 17)	*p*-Value
FSH (IU/L)	5.1 (2.5–6.2)	4.8 (3.6–5.6)	0.692
LH (IU/L)	2.0 (1.0–3.9)	5.1 (3.6–6.3)	0.010
Estradiol (nmol/L)	0.08 (0.07–0.24)	0.21 (0.14–0.33)	0.090
Testosterone (nmol/L)	0.83 (0.62–1.10)	1.00 (0.81–1.30)	0.058
SHBG (nmol/L)	56.0 (46.3–67.0)	65.0 (48.5–92.0)	0.121
TSH (IU/L)	1.55 (1.23–2.20)	2.30 (1.50–2.90)	0.061
fT4 (pmol/L)	12.0 (11.0–14.0)	13.0 (11.5–13.5)	0.741
Glucose (mmol/L)	4.7 (4.6–5.0)	4.8 (4.7–4.9)	0.283
HbA1c (mmol/L)	29.0 (28.0–33.0)	29.0 (28.0–31.0)	0.714
Insulin (pmol/L)	29.0 (22.8–40.8)	41.0 (34.5–58.5)	0.022
C-peptid (pmol/L)	463 (402–511)	529 (442–667)	0.052
HOMA-IR	0.545 (0.417–0.765)	0.760 (0.640–1.095)	0.025

Values are expressed as median (25th–75th percentiles). *p*-values refer to difference between groups.

## Data Availability

The data present in this study are openly available in the Supplemental Information as Supplementary Table S1. Raw data for age, height, and weight are not included to ensure anonymity for the athletes.

## Supplementary Materials

The following supporting information can be downloaded at: https://www.mdpi.com/article/10.3390/sports10030037/s1, Supplementary Table S1: Raw data. CSV file containing the raw data from the research.
